# Genetic Mapping Identifies Consistent Quantitative Trait Loci for Yield Traits of Rice under Greenhouse Drought Conditions

**DOI:** 10.3390/genes11010062

**Published:** 2020-01-05

**Authors:** Niranjan Baisakh, Jonalyn Yabes, Andres Gutierrez, Venkata Mangu, Peiyong Ma, Adam Famoso, Andy Pereira

**Affiliations:** 1 School of Plant, Environmental and Soil Sciences, Louisiana State University Agricultural Center, Baton Rouge, LA 70803, USA; jyabes@agcenter.lsu.edu (J.Y.); AGutierrezViveros@agcenter.lsu.edu (A.G.); 77.ramana@gmail.com (V.M.); pma@agcenter.lsu.edu (P.M.); 2 Rice Research Station, Louisiana State University Agricultural Center, Crowley, LA 70578, USA; afamoso@agcenter.lsu.edu; 3 Department of Crop and Soil Sciences, University of Arkansas, Fayetteville, AR 72701, USA; apereira@uark.edu

**Keywords:** drought, grain yield, greenhouse, panicle, QTL, rice

## Abstract

Improving drought resistance in crops is imperative under the prevailing erratic rainfall patterns. Drought affects the growth and yield of most modern rice varieties. Recent breeding efforts aim to incorporate drought resistance traits in rice varieties that can be suitable under alternative irrigation schemes, such as in a (semi)aerobic system, as row (furrow-irrigated) rice. The identification of quantitative trait loci (QTLs) controlling grain yield, the most important trait with high selection efficiency, can lead to the identification of markers to facilitate marker-assisted breeding of drought-resistant rice. Here, we report grain yield QTLs under greenhouse drought using an F_2:3_ population derived from Cocodrie (drought sensitive) × Nagina 22 (N22) (drought tolerant). Eight QTLs were identified for yield traits under drought. Grain yield QTL under drought on chromosome 1 (phenotypic variance explained (PVE) = 11.15%) co-localized with the only QTL for panicle number (PVE = 37.7%). The drought-tolerant parent N22 contributed the favorable alleles for all QTLs except *qGN3.2* and *qGN5.1* for grain number per panicle. Stress-responsive transcription factors, such as ethylene response factor, WD40 domain protein, zinc finger protein, and genes involved in lipid/sugar metabolism were linked to the QTLs, suggesting their possible role in drought tolerance mechanism of N22 in the background of Cocodrie, contributing to higher yield under drought.

## 1. Introduction

The onset of widespread climate change is causing erratic rainfall patterns, leading to limited availability of surface water for irrigation in field crops, thus creating a water deficit or drought scenario in arid and semi-arid regions [1]. Rice is life to billions of people who depend on it as their primary source of calories. Rice production needs to be doubled in the next decade to meet the demand of the ever-increasing rice-consuming population. This has to be achieved with less land and without exhausting natural resources, including water [2]. Drought is considered the most devastating abiotic stress for rice, causing up to 50% yield loss worldwide [3]. Most modern high-yielding rice cultivars are drought sensitive, and the impact on yield is severe when the plants experience drought at the reproductive stage [4]. Therefore, it is important to breed drought-resistant rice varieties that are suitable for rainfed areas, which occupy almost half of the rice growing areas.

In the U.S., rice has historically been cultivated under irrigation because water is abundant, especially in Louisiana. However, freshwater availability can be affected by storm surge, which affects the quality of surface irrigation water by making it saline or alkaline. So, considering the importance of the shortage of quality ground and surface water available for irrigation, alternative water management strategies, such as alternate wetting and drying and furrow-irrigation (row rice) are being adopted as promising strategies to tackle future water shortages. Additionally, row rice production practices are gaining popularity as this practice requires fewer resources in field preparation and provides farmers with more flexibility when deciding which crop that they will produce. Therefore, efforts are underway for the development of drought-tolerant rice varieties with high water use efficiency that can fit to the alternate water management schemes, such as aerobic conditions [5].

The development of successful aerobic rice cultivars could be achieved by combining the high-yielding traits of irrigated rice with the drought-tolerant traits of traditional upland rice cultivars. Secondary drought resistance traits, such as relative water content, membrane stability index, leaf area, canopy temperature, root growth, etc., are difficult to quantify for their direct contribution to grain yield in a breeding population. On the other hand, grain yield traits under drought were considered to be consistent and most effective for the selection of drought-resistant rice genotypes [4,6].

Identification and introgression of quantitative trait loci (QTLs) controlling grain yield under drought are an effective approach to breeding high-yielding drought-resistant rice [7,8,9]. To this end, 16 grain yield under drought (GYD) QTLs on all but three rice chromosomes [5,7,8] were reported [10]. However, a meta-QTL study identified 14 MQTLs on seven chromosomes including two on chromosome 8 for GYD [11]. The *qDTY12.1* on chromosome 12 was the first QTL reported for grain yield under drought that is consistent over multiple generations [12], and it has been used to develop drought-resistant upland and lowland rice with increased grain yield [13,14]. The QTL *qDTY1.1* on chromosome 1 was identified where drought-resistant varieties Nagina 22 [15,16] and Dhagaddeshi [17] contributed the favorable allele for GYD. Two other QTLs, *qDTY2.3* [18,19] and *qDTY3.2* [20], from the variety Vandana interact with *qDTY12.1* to enhance yield and harvest index under severe upland and lowland drought conditions [21]. Five GYD QTLs on chromosomes 1, 6, 8, 10, and 12 were identified [22], with the largest effect *qDTY12.1* coinciding with a minor QTL for grain thickness [23].

Considering the narrow genetic base and drought sensitivity of the U.S. rice germplasm [24], introgression of drought-tolerant genes from drought-tolerant germplasm have been initiated into the background of rice varieties adapted to the southern U.S. [5,25]. Previously, we reported six QTLs (three on chromosome 1 and one each on chromosome 5, 8, and 9) contributing to grain yield under controlled greenhouse conditions, where the favorable alleles for four QTLs were contributed by the drought-tolerant donor variety Vandana [25]. Here, we report on the identification of genetic determinants for grain yield traits under greenhouse drought conditions in the same U.S. genetic background Cocodrie but with a different drought-resistant donor, Nagina 22.

## 2. Materials and Methods

### 2.1. Mapping Population

The population used in the present mapping study included 190 F_2:3_ progeny lines derived from the F_1_s between a US-bred drought-sensitive variety ‘Cocodrie’ [26] and an “Aus”-type Indian-origin drought-resistant Nagina 22 (N22). N22 is a short-duration (90–95 days), deep rooted, drought- and heat-tolerant “Aus” rice landrace [27].

### 2.2. Drought Screening and Phenotypic Data Analysis

Phenotyping of 190 F_2:3_ progeny lines and the parents for their drought response was done inside the Louisiana State University Agricultural Center’s greenhouse at Gourrier Ln, Baton Rouge, LA during spring 2015 and fall 2017 as previously described [25,28]. Briefly, two sets of six plants per line including parents were grown in 2.8 L plastic pots with bottom holes under normal irrigation. For drought stress, irrigation was withdrawn from one set of 45-day-old plants for two weeks (soil moisture content ~0.07 m^3^/m^3^), while the other set was well watered (control; soil moisture content 0.48 m^3^/m^3^). Following drought, irrigation was resumed until grain maturity. The experiment was conducted in a complete randomized block design, with three replications in ceramic trays (blocks), as described earlier [25]. Data were recorded on both control and recovered plants for yield traits such as the number of panicles per plant, the number of grains per panicle, and grain yield (g) per plant.

Phenotypic data were analyzed using the basic R v3.4.1 package and SAS 9.3 [29] as described earlier [25,30]. Normality of the data was determined by the Shapiro–Wilk test and Pearson’s test was conducted to estimate the correlations among the yield traits [30]. Analysis of variance (ANOVA) of the yield traits was estimated using mixed model (Proc MIXED). Broad-sense heritability was calculated on a family means basis using ANOVA-derived variance components. Frequency distribution of the yield traits under drought was charted as histogram using basic R v3.4.1 package.

### 2.3. Molecular Markers and Genotyping

Markers used for genotyping consisted of 134 SSRs [31], four Indel markers [32], and six genic SSRs [25] that were polymorphic between Cocodrie and N22. In addition, eight polymorphic SNPs were used to narrow down the gaps in the QTL regions in chromosome 1, 8, and 11.

### 2.4. Genotyping, Linkage and QTL Mapping

Genotyping of the mapping population with the SSR and Indel markers was conducted on single F_2_ plants following Solis et al. [25]. SNP genotyping was performed using KASP markers on an LGC SNP genotyping platform following the manufacturer’s instructions (https://biosearch-cdn.azureedge.net/assetsv6/KASP-genotyping-chemistry-User-guide.pdf).

Multipoint linkage analysis was performed using ICIM software v 4.0 [33], using a recombination frequency (r) set at 0.45. The map position (cM) of markers was estimated using the Kosambi mapping function and ordered with a threshold logarithm of odd (LOD) set at 3.0. QTL analysis with the mean data on yield and yield attributing traits of the F_2:3_ progeny lines averaged over two years was conducted by interval mapping (IM) and inclusive composite interval mapping (ICIM). QTLs explaining ≥5% phenotypic variance with LOD ≥ 2.5 were declared significant, and QTL nomenclature followed Solis et al. [25]. Genotypic frequency was calculated for yield trait loci using the marker closest to the QTL peak.

### 2.5. The Identification of Candidate Genes in QTL Region

Physical positions of the marker closest to a QTL was retrieved from Gramene (www.gramene.org) and inputted to identify the genes using SNP-Seek II [34]. Genes were compared to the set of genes that were significantly differentially expressed from the transcriptome study using N22 [35] and Vandana [25].

## 3. Results

### 3.1. Genetic Variation for Grain Yield and Yield Traits under Drought

**Figure 1:**
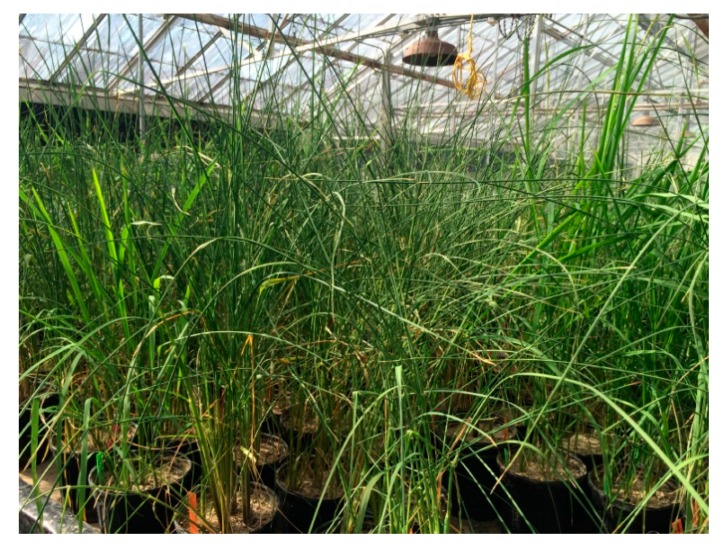
F_2:3_ progenies derived from Cocodrie × Nagina 22 (N22) showing segregation for drought response phenotypes under greenhouse conditions.

**Figure 2:**
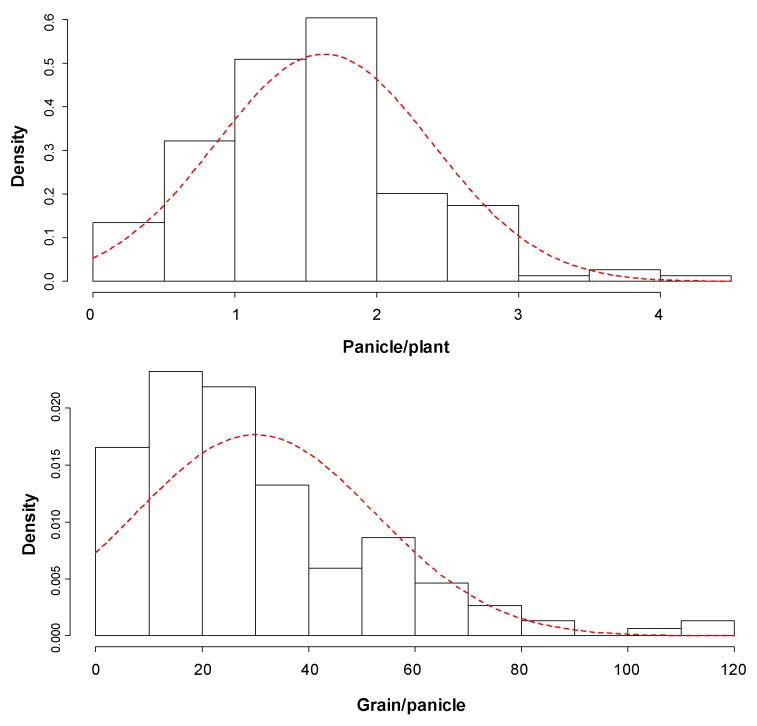

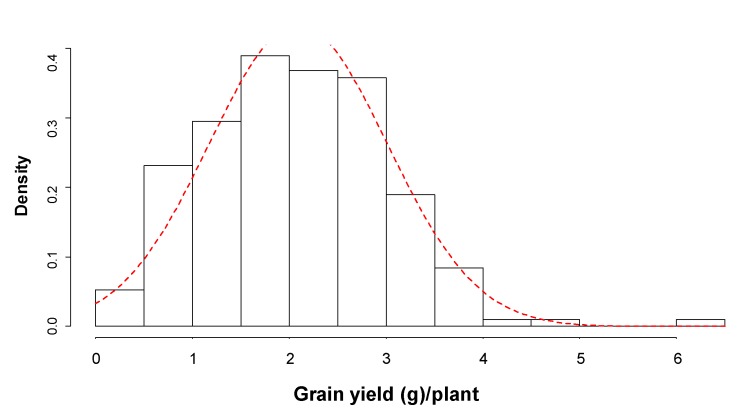
Frequency distribution of yield traits (panicle/plant—upper panel; grain/panicle—middle panel; and grain yield (g)/plant—lower panel) of the F_2:3_ progenies derived from Cocodrie × N22 under greenhouse drought conditions. Density = frequency/interval. Frequency = area of the bar representing the number of F_2:3_ lines in a class.

The Cocodrie × N22 F_2:3_ progenies showed variation in their phenotypic response such as leaf rolling, drying and wilting symptoms to drought stress (Figure 1). The average panicle number per plant, grains per panicle, and grain yield per plant of the stressed F_2:3_ lines were 1.7 (0.14–9.00), 30 (0.50–115.25), and 2.02 g (0.35–4.60), respectively (Supplementary Table S1). The parents, Cocodrie and N22 had contrasting responses under drought, where panicle number, grain number and grain yield per plant were 2.2, 44 and 2.6 g, respectively, for Cocodrie, and 8.4, 93 and 12.1 g, respectively, for N22 (Supplementary Table S1). The phenotypic distribution of all the yield traits studied under drought showed a (near)normal distribution (Figure 2) with *p*-values 0.041, <0.001 and <0.0001, and W = 0.9964, 0.9617 and 0.9913, respectively, for panicle number, grain number and grian yield (g).

The yield traits showed a significantly positive correlation among themselves (Supplementary Table S2). The correlation between grain yield per plant and grain number per panicle was significantly high (0.89, *p* < 0.01) followed by that between grain yield and panicle number per plant (0.69, *p* < 0.01). However, the correlation between panicle number and grain number was moderate (0.34), yet significant (*p* < 0.01). The broad sense heritability (*H*^2^) for panicle number, grain number, and grain yield was moderately high at 0.68, 0.55, and 0.32, respectively.

### 3.2. QTLs Controlling the Yield Traits

Linkage analysis of 152 markers generated a 1888.6 cM-long map, where the average distance between the adjacent markers was 15.1 cM (Supplementary Figure S1). Chromosome 3 was the longest and chromosome 10 was the smallest. The chromosomes had markers distributed over the entire length with some gaps, especially in chromosome 3, 4, 5, 6, 7 and 12. Chromosome 6 had the largest gap of ~22.6 Mbp between RM589 and RM162. The gaps in the chromosomal region were due to the lack of polymorphic markers identified between the parents.

Altogether, eight QTLs were identified by the ICIM for the yield traits under drought stress (Table 1; Supplementary Figure S1). A major QTL on chromosome 1 (*qPN1.1*) was found to control panicle number explaining 37.7% of the total phenotypic variance with the highest LOD (15.1). The QTL was delimited by markers SNPID280 and RD105_2, with a positive additive genetic variance of 2.6, suggesting that N22 contributed the favorable allele.

Three QTLs, two on chromosome 3 (*qGN3.1* and *qGN3.2*) and one on chromosome 5 (*qGN5.1*), were detected for grain number per panicle. Coincidentally, the phenotypic variance explained by each QTLs were 3.8% each. While N22 contributed the favorable alleles for qGN3.1, the drought-sensitive parent, Cocodrie contributed the positive alleles for qGN3.2 and qGN5.1.

Four QTLs, one each on chromosome 1 (*qGY1.1*), 7 (*qGY7.1*), 8 (*qGY8.1*) and 11 (*qGY11.1*), which collectively controlled 45% of the phenotypic variance, were identified for grain yield under drought by ICIM. Individually, *qGY8.1* explained the maximum phenotypic variance (13.3%) followed by *qGY7.1* (12.7%), *qGY1.1* (11.1%) and *qGY11.1* (7.9%). For all the QTLs, the alleles for increasing mean grain yield were contributed by the drought-resistant parent, N22. QTLs, *qGY1.1* and *qPN1.1* were co-localized.

In addition to the additive QTLs, six epistatic QTLs were discovered from ICIM (LOD > 5.0) controlling panicle number and grain yield under drought (Supplementary Table S3). Four inter-chromosomal epistatic QTLs were responsible for controlling panicle number with the highest phenotypic variance explained (PVE) (10.1%) by the interaction of QTLs on chromosome 2 and 12. Two epistatic QTLs, one between chromosome 1 and 3 and the other between chromosome 9 and 12 controlled grain yield with equal contribution to the PVE (6.5%). Although an epistatic QTL was identified on chromosome 1, none of the epistatic QTLs overlapped with the additive QTLs.

### 3.3. Allelic Contribution at the Drought Yield Traits QTLs

Genotype frequency of the F_2:3_ lines calculated for the markers closest to the yield traits QTLs showed that the average number of panicles and grain yield of the lines homozygous for the N22 allele at three flanking markers (SNP280, RM457, and RM10) was higher than the homozygous Cocodrie allele (Supplementary Figure S2). On the other hand, the frequency of Cocodrie allele (0.098) and Cocodrie/N22 alleles (0.090) for mean grain yield of the lines were nearly equal at marker closest to qGY11.1 (SNPID202). Lines homozygous with N22 alleles and/or heterozygous at CVSSR21 had higher grains per panicle whereas lines homozygous for Cocodrie allele and/or heterozygous at RM1278 and RM5755 had higher grain number per panicle.

### 3.4. Genes Underlying QTL Regions

Comparison of the genes identified based on the physical location of the markers against known drought responsive genes of N22 and Cocodrie [25,35] showed several genes with known/unknown functions and transposons underlying the QTLs for yield traits under drought stress. Phosphatidylserine decarboxylase DUF630/DUF632 domains containing protein, serine-threonine protein kinase, APETALA2/ethylene-responsive binding protein, trehalose 6-phosphate phosphatase (TPP), aquaporin protein TIP1.2, WD40 (G-β) repeat domain containing protein, purple acid phosphatase, and zinc finger protein are some of the stress-responsive genes that are closely linked (within 10 Kb distance) to the marker closest to the peak of the yield traits QTLs (Supplementary Table S4).

## 4. Discussion

Drought is an increasing threat to the sustainability of rice production worldwide due to irregular rainfall patterns under the climatic uncertainties. The recent shifts in climate have challenged rice researchers to devise scientific strategies to address this issue, such as to breed new rice varieties that can adapt to periodic dry spells even in areas where water availability for irrigation is considered to be abundant. QTL mapping strategy has been widely used to understand the genetic complexity of quantitative traits such as drought tolerance in rice. Earlier studies suggested direct selection for grain yield as the most effective approach to breeding improved drought-resistant rice varieties [19,25,36]. Many previous studies have identified QTLs for grain yield traits under both vegetative- and reproductive-stage drought stress through selective/genome-wide genotyping [12,14,15,16,17,18,25,37,38,39,40]. The present study was undertaken to identify genomic regions and the genes that govern rice yield traits under drought in elite US rice germplasm. In the absence of a rain-out shelter to control rainfall under natural conditions, the mapping population was evaluated under controlled greenhouse conditions that can circumvent the variation due to micro (soil) and macro environmental factors [25]. Differential drought response of the mapping population and the parents, Cocodrie and N22, was evident from the phenotypes, such as leaf rolling, leaf drying, and yield metrics (Figure 1; Supplementary Table S1). The moderate broad sense heritability values for yield traits (Supplementary Table S1) presented in this study are in agreement with other studies reported for yield traits under controlled drought conditions [25,41].

A total of eight QTLs that control yield traits under drought response were identified in chromosomes 1, 3, 5, 7, 8, and 11 in the present study. However, none of the 16 QTLs [10] and seven meta-QTLs [11] reported earlier were identified in chromosome 7 and 8. QTLs associated with yield traits are often co-localized [14,19,42], and in the present study a single QTL on chromosome 1 (SNPID280 and RD0105_2) contributed to both panicle number and grain yield under drought. However, QTLs for drought tolerance secondary traits could also contribute to the yield under drought [6].

The marker SNPID280 associated with QTLs *qGY1.1* and *qPN1.1* on chromosome 1 (42.32 Mbp) for grain yield and panicle number, respectively, under drought, in our study was co-localized with the known grain yield QTLs reported earlier [15,16]. The chromosomal region harboring *qGY1.1* also harbors the *sd1* gene for semi-dwarf plant height, suggesting the relevance of the ‘green revolution gene’ in drought stress response in rice [43,44]. The same genomic region harbors the single major QTL (37.732% PVE) identified for panicle number (productive tillers) under drought. However, *qGY1.1* did not overlap with the grain yield QTL reported earlier on chromosome 1 in a population derived from Cocodrie and Vandana [25] where favorable alleles were contributed by the susceptible parent Cocodrie in contrast to N22 in the present study. Such observations underscore the importance of genetic background in favorable allele discovery. Due to high positive correlation between the yield components such as panicle number and grain yield under drought, and negative correlation with plant height, QTLs for different yield-related traits co-localize in the same or closely linked chromosomal regions [44].

The grain yield QTL, *qGY7.1* (RM 10–RM47) identified on chromosome 7 in this study spanned *qYP7.2* (RM1377–RM1279), the QTL reported for grain yield per plant under drought [45]. Chromosome 7 was also found to contain a QTL for the yield attributing trait, spikelet fertility from a greenhouse drought study involving an independent set of population from the same parents [16]. The QTL on chromosome 7, *qGY7.1* identified in this study was not discovered in the Cocodrie × Vandana population that we reported earlier [25].

The QTL on chromosome 8 (RM22926–SNPID457) explained the highest phenotypic variance (13.26%) for grain yield (Table 1). This QTL was co-localized with the yield QTL under greenhouse drought conditions that we reported earlier using the population derived from Cocodrie and Vandana [25]. The genomic region also overlapped with the metaQTL *MQTL8.2* [11]. The QTL, *qGY8.1* for grain yield under aerobic conditions [46] and *qDTY8.1* under drought stress [47] are co-localized between RM339 and RM210, close to regions harboring QTLs for other yield related traits and root length [46]. Genome-wide association studies also identified the marker RM6070 of chromosome 8 to be significantly associated (*p* < 0.01) with both plant height (R^2^ = 3.96) and percentage seed set (R^2^ = 12.85%) [48]. A major QTL located on chromosome 8 explaining 54% of the phenotypic variance for grain yield was reported in Swarna × *Oryza nivara* population [49]. Thus, chromosome 8 appears to be a hot spot for alleles with positive effects on yield traits under drought. However, the QTL peak detected in the present study did not co-localize with the QTLs reported on chromosome 8 earlier by Prince et al. [50] under target environments.

The QTL *qGY11.1* on chromosome 11 (SNPID452–SNPID202) that explained 7.86% of the variance for grain yield has not been reported earlier for grain yield under drought. However, a QTL *qSF11.19* (19.37 Mbp) [16] identified to control spikelet fertility under drought stress is located ~4 Mbp apart from the SNPID452 (23.9 Mbp). On the other hand, a QTL for deep root length under drought was observed between 9.0 Mbp (RM202) and 18.4 Mbp (RM229) on chromosome 11 [51] in the proximity of *qGY11.1*.

Favorable alleles for the number of grains per panicle were contributed by the drought-resistant parent N22 for *qGN3.2* (RM514–RM5755) as well as the drought-sensitive parent Cocodrie for *qGN3.1* (RM1278–RM1867). The QTL *qGN3.2* covered the metaQTL *MQTL3.2* reported for grain yield under stress [11]. *MQTL3.1* (1.3 Mbp) and a QTL at RM232 (1.0 Mbp) for single plant yield under drought stress [52] were also found close to the grain number QTL *qGN3.1* (4.5 Mbp) identified in the present study. However, no QTL for yield traits under drought was identified on chromosome 3 in managed stress and target environments [50] and in our previous controlled drought treatment study [25]. However, a QTL at 33.1 Mbp for % plant dry matter content [16] and QTLs for total shoot dry weight, leaf/stem dry weight, deep root length at RM520 (30.9 Mbp) were reported nearby RM514 (35.2 Mbp) [51] delimiting *qGN3.2*.

The region between RM440–CVSSR21 on chromosome 5 was also found to be consistent for yield traits under drought stress. It harbored *qGN5.1* for the number of grains per panicle in the present study whereas it directly controlled the grain yield in our previous study [25]. Wang et al. [53] identified four QTLs between S4134205–S7643153 of chromosome 5 that controlled grain yield and related traits in two genetic backgrounds and different environments whereas Yue et al. [54] identified a minor QTL for test weight under drought between RM509–RM430. However, none of these regions overlapped with *qGN5.1* or *qGY5.1* [25].

In contrast to the *QTL12.1* for high grain yield under drought that was identified in the F_3_ population of Vandana/Way Rarem and contributed by the drought-sensitive parent Way Rarem [12], none of the QTLs in the present study were identified on chromosome 12 for grain yield under drought. On the other hand, consistent with our previous report [25], a QTL for grain yield was identified under non-stressed (well-watered) control condition, where the favorable allele was contributed by the drought-sensitive parent Cocodrie (Supplementary Figure S1). Such observations were also documented before, where the QTL region reported for grain yield under drought on chromosome 8 [11] overlapped *QTL8.1* (RM337–RM3664; peak RM8020) that was associated with grain yield under well-watered upland conditions [12].

Most genes identified underlying the QTL regions are known to be responsive to abiotic stress, especially drought. While TPP (*LOC_Os02g548200*) and serine-threonine protein kinase (*LOC_Os06g18820*) were not differentially expressed in N22 relative to IR64 [35], Solis et al. [25] reported a 2.53-fold and 5.45-fold increase in their expression in the drought-tolerant variety Vandana relative to the drought-sensitive variety Cocodrie. However, there was no change in expression of phosphatidylserine decarboxylase (*LOC_Os01g72940*), WD40 protein (*LOC_Os03g08830*), and zinc finger protein (*LOC_Os03g08840*) in either N22 or Vandana under stress relative to the drought-sensitive varieties. DUF630/DUF632 domains containing protein (*LOC_Os01g72970*) and EREB (*LOC_Os08g31580*) were upregulated in N22 by 4.37-fold and 1.47-fold, respectively, under desiccation stress [35]. Recently, there are reports of genes linked to QTLs expressed under drought stress [16,25,50]. The genes and associated gene networks identified under stress will enhance our ability to understand drought response mechanisms and utilize the knowledge to improve grain yield under drought stress.

Controlled drought stress treatment in potted plants under greenhouse conditions could circumvent the problems associated with field conditions, such as variation in soil texture, soil temperature, and other environmental factors, such as humidity, disease and insect pressures that confound phenotyping and consistent QTL detection. However, grain yield QTLs under controlled greenhouse drought conditions may not be expressed under target field environments [55] as plant traits will vary in their response with varying timing and severity of drought under the rainfed rice ecosystem [50,56,57]. For example, there may not be significant correlation between QTLs for drought tolerance traits in upland and low land rice varieties [58] due primarily to the change in hydrology where soil transitions from flooded and anaerobic to drought and aerobic [59]. However, large-effect QTLs, such as deeper rooting 1 (*DRO1*) that confers drought resistance in paddy fields with enhanced yield [60] was identified under controlled conditions drought [61]. Further, consistency in the expression of the major-effect grain yield QTL on chromosome 1 under field drought conditions [15,17] and greenhouse drought conditions in the present study and earlier reports [16,25] suggest that in the absence of a rain-out shelter, precise maintenance of the soil moisture content for (controlled) greenhouse drought conditions could successfully be exploited for the identification of QTL regions controlling grain yield under stress. In addition to chromosome 1, QTLs on chromosome 5 and 8 identified in the present study and our previous study [25] under greenhouse drought conditions indicate that these QTLs will also most likely be expressed under drought field conditions.

A successful marker-assisted breeding to improve grain yield under drought will depend on the identification and consistent expression of large-effect QTLs under natural drought conditions in various target field environments [18,62]. To this end, we are currently evaluating our advanced generation recombinant inbred lines under controlled field drought conditions and/or aerobic conditions to validate the consistency of identified yield traits QTLs and to precisely identify causal genes for the subsequent development of diagnostic markers. Our present research represents findings from the ongoing efforts to develop rice varieties that will have little to no yield penalty under periodic dry spells (such as aerobic conditions) in furrow-irrigated (row) rice in southern U.S., such as in Louisiana, Arkansas, Texas, and Mississippi.

## Figures and Tables

**Table 1:** Quantitative trait loci (QTL) identified for yield traits in F_2:3_ progenies derived from Cocodrie × N22 under greenhouse drought.

Trait	QTL	Chr	Position (cM)	Left Marker	Right Marker	LOD	PVE	Add	Dom	Left CI	Right CI
Panicle/plant											
	*qPN1.1*	1	279.6078	SNPID280	RD0105_2	15.104	37.732	2.566	−2.6217	276.6577	282.558
Grain/panicle											
	*qGN3.1*	3	31.4001	RM1278	RM1867	5.956	3.824	21.694	−21.3334	20.95	43.0499
	*qGN3.2*	3	130.6988	RM514	RM5755	4.339	3.753	−21.515	−21.0113	119.8487	140.4494
	*qGN5.1*	5	107.2989	RM440	CVSSR21	3.354	3.770	−21.117	−22.9362	104.549	114.1488
Grain yield/plant											
	*qGY1.1*	1	287.7083	SNPID280	RD0105_2	2.787	11.147	0.983	−1.395	282.7581	294.8588
	*qGY7.1*	7	113.8988	RM10	RM47	2.661	12.652	0.370	−1.0751	98.5491	124.2487
	*qGY8.1*	8	30.6001	RM22926	SNPID457	5.777	13.264	0.424	0.2553	22.8501	37.65
	*qGY11.1*	11	111.7989	SNPID452	SNPID202	3.767	7.859	0.396	0.0759	105.349	118.3488

Chr = chromosome; cM = centi Morgan; PVE = phenotypic variance explained; Add = additive; Dom = dominance; CI = confidence interval.

## Supplementary Materials

The following are available online at https://www.mdpi.com/2073-4425/11/1/62/s1. Figure S1: Linkage maps of F_2:3_ progenies derived from Cocodrie × N22. Quantitiative trait loci (QTLs) controlling yield traits under greenhouse drought and control conditions are shown on chromosomes 1, 3, 5, 7, 8, 9, 11 and 12. QTLs in green, blue and red represent genomic regions associated with panicle number, grain number, and grain yield, respectively, under drought conditions. QTLs in orange and purple are associated with grain number and grain yield, respectively, under non-stress control. Figure S2: Genotype frequency of the F_2:3_ progenies derived from Cocodrie × N22 at the marker closest to the genomic regions associated with grain yield traits under greenhouse drought conditions. A, Cocodrie; H, heterozygous; B, N22. Table S1: Yield traits of F_2:3_ progenies derived from Cocodrie × N22 under greenhouse drought. Table S2: Correlation between yield traits of F_2:3_ progenies derived from Cocodrie × N22 under greenhouse drought. Table S3: Epistatic quantitative trait loci controlling panicle number and grain yield of F_2:3_ progenies derived from Cocodrie × N22 under greenhouse drought. Table S4: Genes within 10 Kb of the marker closest to the QTL peak for the yield traits.
