# Supplementary material for: Genetic Mapping Identifies Consistent Quantitative Trait Loci for Yield Traits of Rice under Greenhouse Drought Conditions
Source: Genes (Basel). 2020 Jan 5;11(1):62. doi: 10.3390/genes11010062 (PMC7017276; doi:10.3390/genes11010062)
Supplement: Supplementary file 1 [file genes-11-00062-s001.pdf]

Supplementary Figure S1

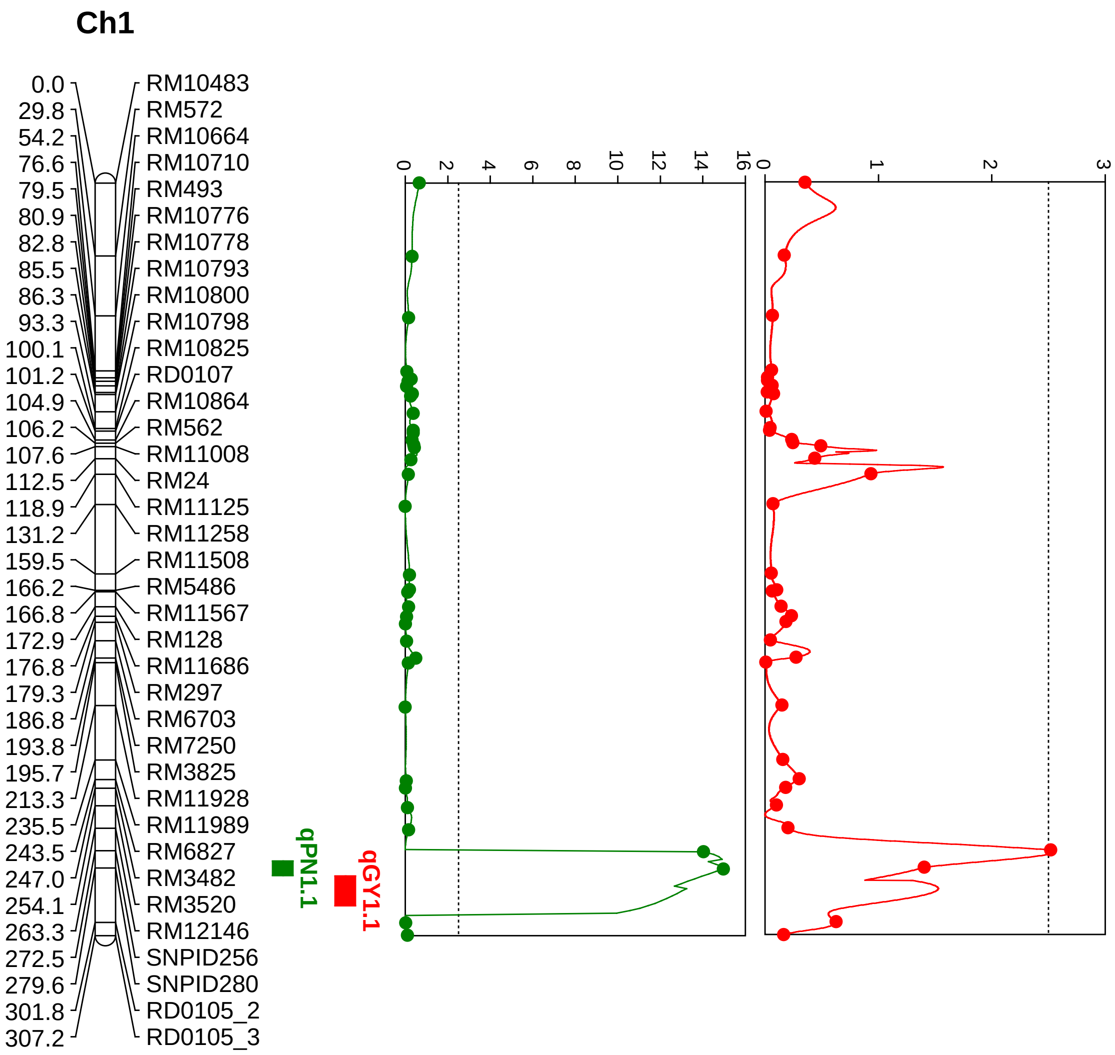

Supplementary Figure S1

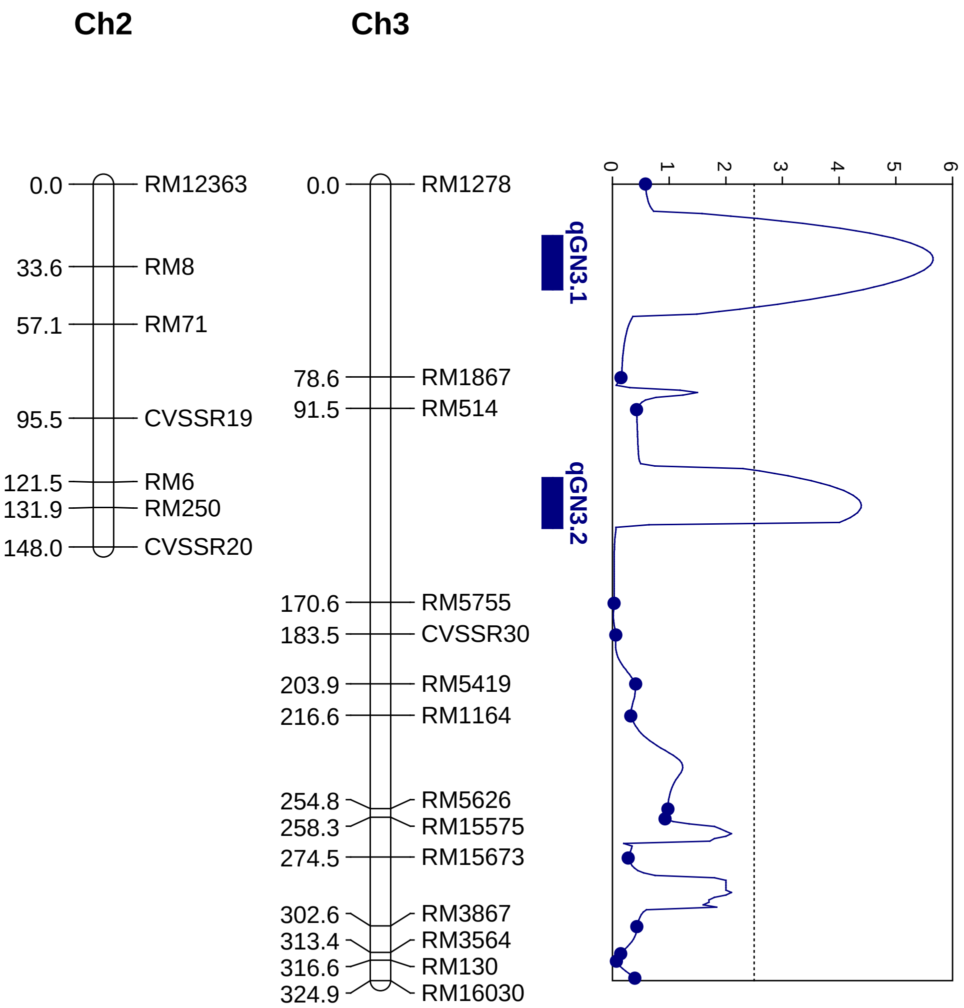

Supplementary Figure S1

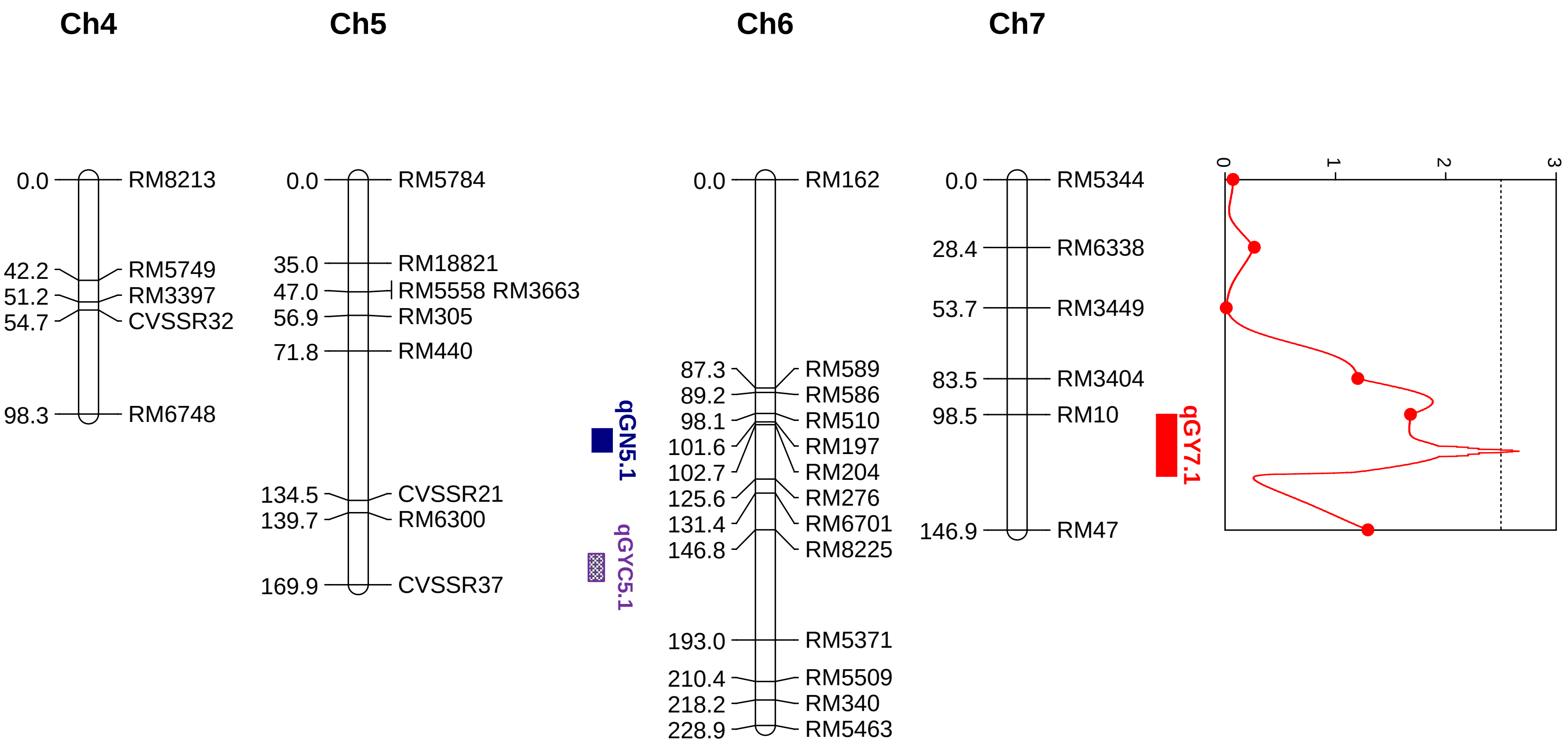

Supplementary Figure S1

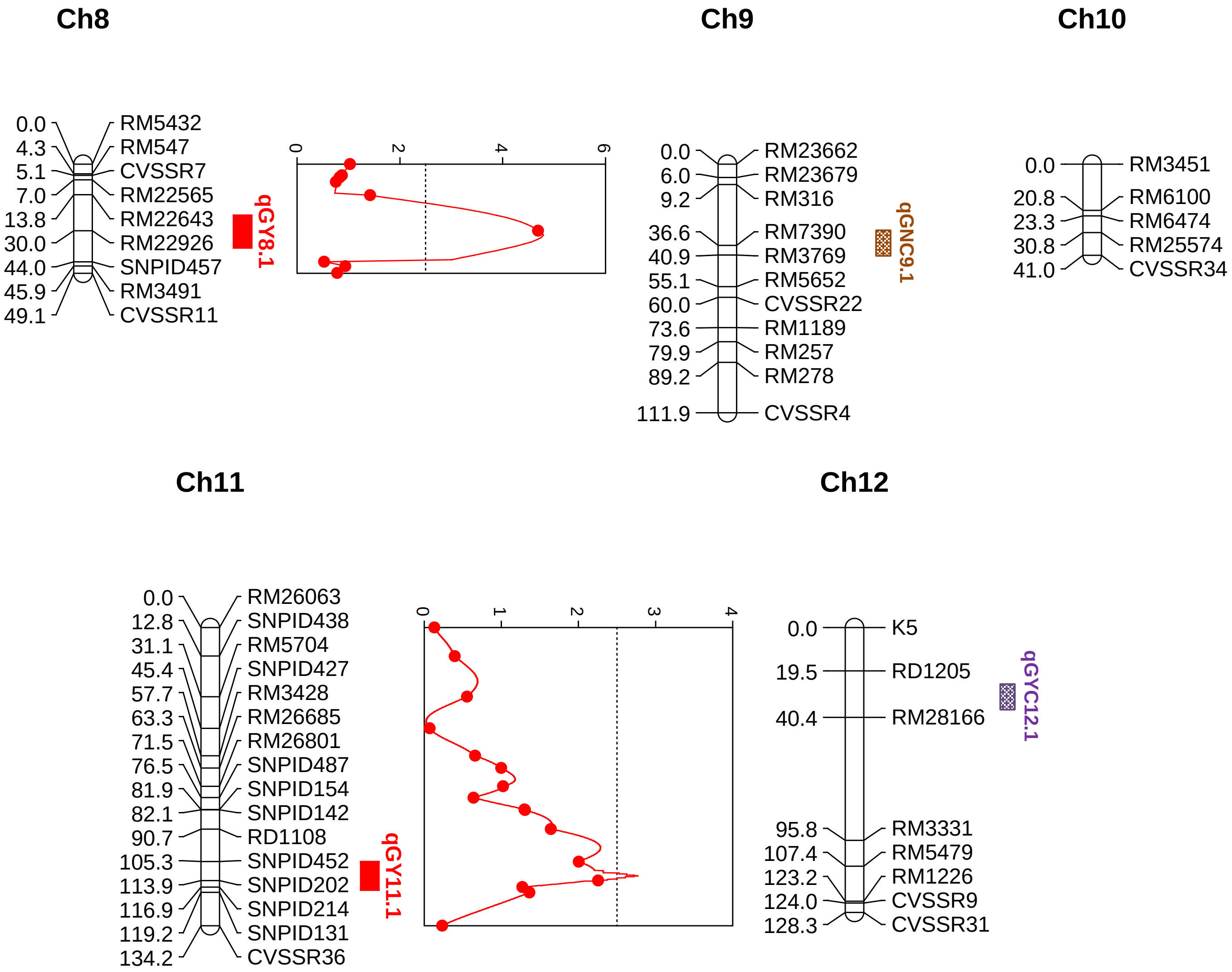

Supplementary Figure S2

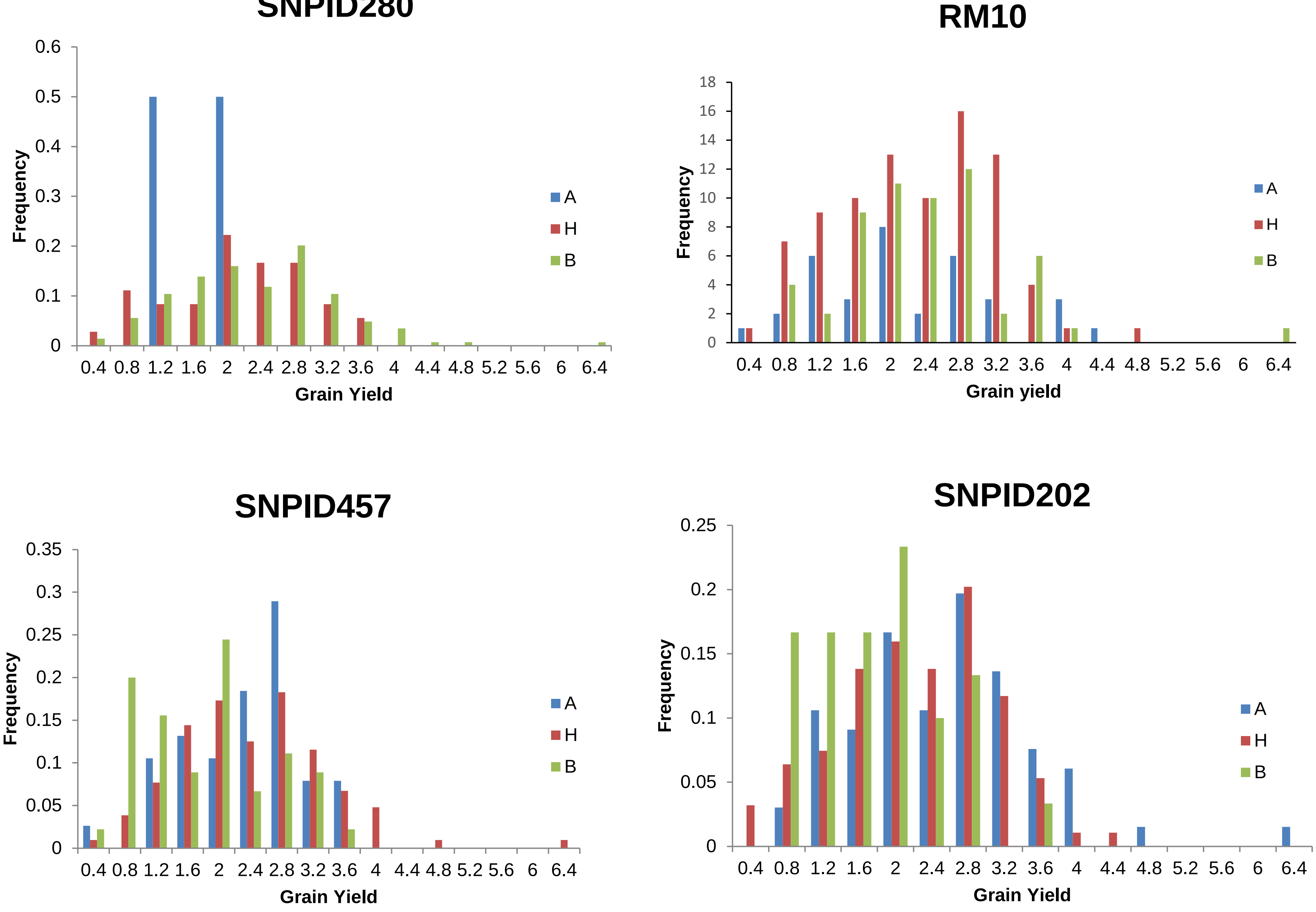

Supplementary Figure S2

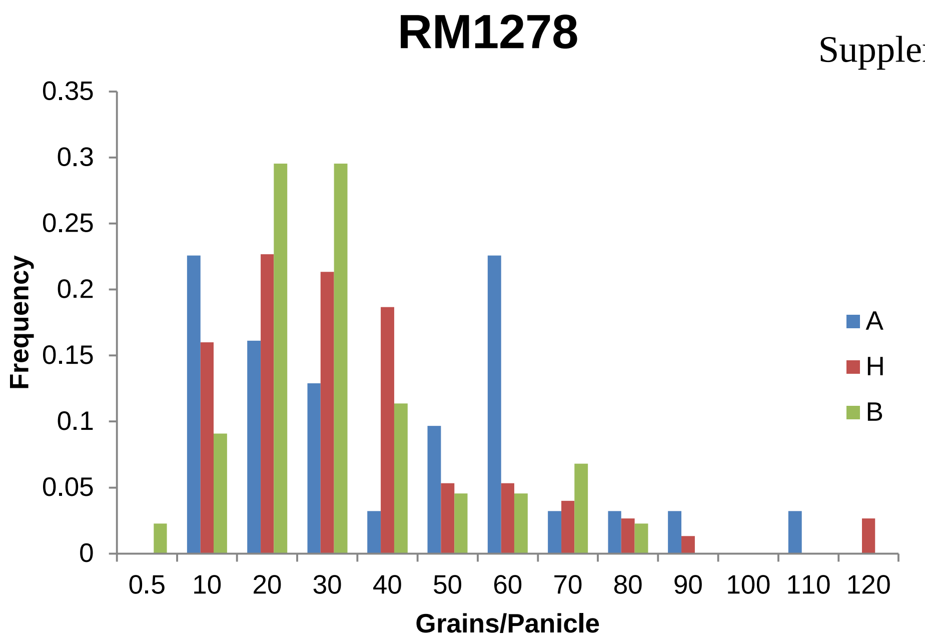

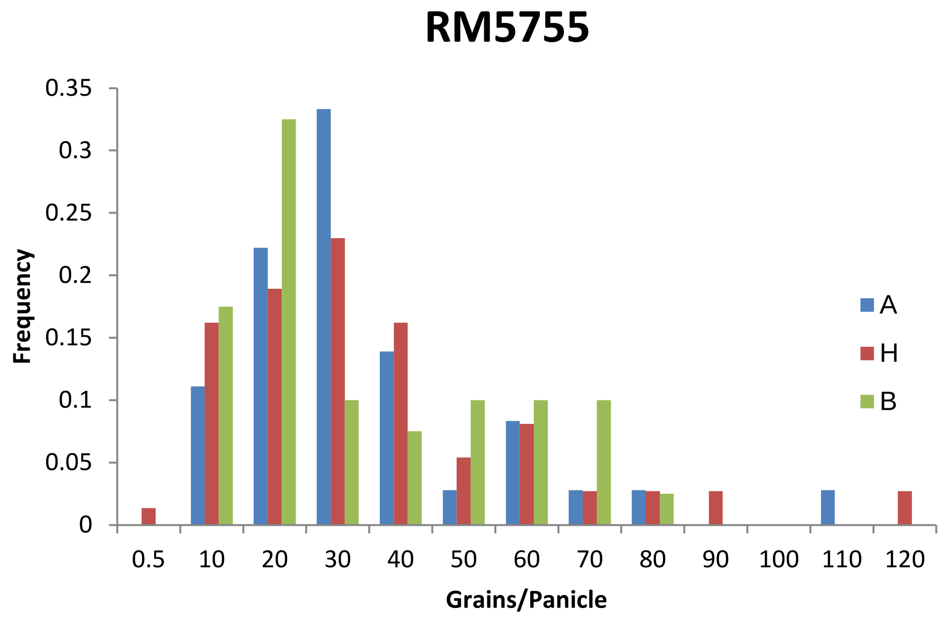

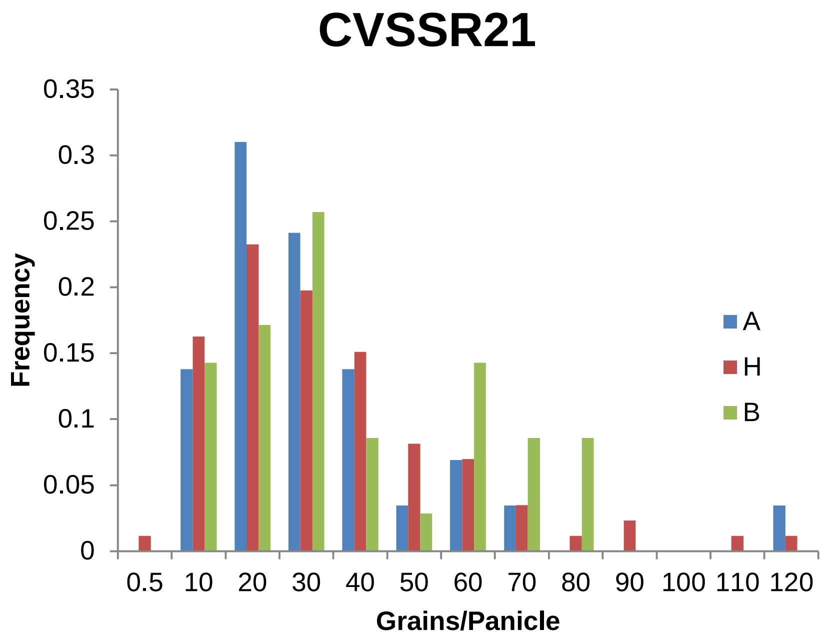

Supplementary Table 1. Yield traits of $F_{2:3}$ progenies derived from Cocodrie x N22 under greenhouse drought

	Parents		RILs		
Trait	Cocodrie	N22	Mean±SD	Range	Broad sense heritability (H^2)
Panicle/plant	2.2	8.4	1.68±0.97	0.14 - 9.00	0.68
Grain/panicle	44	93	29.98±22.60	0.50 - 115.25	0.55
Yield/plant	2.6	12.1	2.02±0.88	0.35 - 4.60	0.32

Supplementary Table 2. Correlation between yield traits of $F_{2:3}$ progenies derived from Cocodrie x N22 under greenhouse drought

**Trait**	Yield/plant	Panicle/plant	Grain/panicle
GY	1	0.69367** (<0.0001)[a]	0.89031** (<0.0001)
PN		1	0.33782** (<0.0001)
GN			1

[a] Values in the parenthesis represent p-values for Pearson correlation

Supplementary Table 3. Epistatic quantitative trait loci controlling panicle number and grain yield of $F_{2:3}$ progenies derived from Cocodrie x N-22 under greenhouse drought

Trait	Ch 1	Pos 1	Left marker 1	Right marker 1	Chr 2	Pos 2	Left marker 2	Right marker 2	LOD	PVE (%)	Add 1	Add 2	Dom 1	Dom 2	Add x Add	Add x Dom	Dom x Add	Dom x Dom
PN	2	60	RM71	CVSSR19	3	270	RM15575	RM15673	5.1	7.3	0.98	0.12	-0.80	-0.93	0.70	-1.05	0.22	1.00
PN	6	150	RM8225	RM5371	8	10	RM22565	RM22643	6.1	6.0	0.37	-0.53	0.12	0.08	-0.43	-0.37	0.91	-0.11
PN	3	240	RM1164	RM5626	9	0	RM23662	RM23679	5.3	7.3	0.42	0.31	0.46	0.36	0.19	-0.83	-0.65	-0.62
PN	2	15	RM12363	RM8	12	120	RM5479	RM1226	5.7	10.1	0.39	-0.40	-1.03	-0.80	-0.11	-0.08	0.50	1.51
GY	1	180	RM297	RM6703	3	35	RM1278	RM1867	5.8	6.5	-0.31	0.42	0.34	0.86	-0.37	0.77	-1.49	-1.02
GY	9	105	RM278	CVSSR4	12	60	RM28166	RM3331	5.6	6.5	0.87	0.15	-0.83	-0.39	0.30	-1.73	0.36	0.94

Supplementary Table S4. Genes within 10 Kb of the marker closest to the QTL peak for the yield traits

Marker/QTL	Physical location start	Physical location end	Locus ID	Gene annotation
SNPID280/qPN1.1/qGY1.1	42311177	42303454	LOC_Os01g72940	phosphatidylserine decarboxylase,
	42312149	42313577	LOC_Os01g72950	NDH-O (NAD(P)H:plastoquinone dehydrogenase complex subunit O)
	42315264	42317679	LOC_Os01g72960	Gamma-secretase aspartyl protease complex
	42325823	42329066	LOC_Os01g72970	DUF630/DUF632 domains containing protein
	42335754	42329246	LOC_Os01g72980	tRNA uridine 5-carboxymethylaminomethyl modification enzyme gidA
	42349399	42336217	LOC_Os01g72990	Similar to Glucose inhibited division protein A
	42355398	42350927	LOC_Os01g73000	copine
	42360654	42358083	LOC_Os01g73005	expressed protein
	42369770	42370365	LOC_Os01g73024	expressed protein
RM1278/qGN3.1	4550864	4559149	LOC_Os03g08820	expressed protein
	4561061	4568647	LOC_Os03g08830	WD domain, G-beta repeat domain containing protein
	4571025	4569624	LOC_Os03g08834	Conserved hypothetical protein
	4573148	4575550	LOC_Os03g08840	zinc finger protein
RM5755/qGN3.2	5941787	5944063	LOC_Os03g11480	expressed protein
	5946380	5955641	LOC_Os03g11490	Conserved hypothetical protein
	5960605	5955623	LOC_Os03g11500	DUF647 domain containing protein
	5964746	5960793	LOC_Os03g11510	mitochondrial carrier protein
	5967188	5973722	LOC_Os03g11520	expressed protein
	5973868	5979082	LOC_Os03g11530	nucleotide pyrophosphatase/phosphodiesterase
CVSSR21/qGN5.1	791711	788617	LOC_Os05g02400	RNA recognition motif containing protein
	791891	794673	LOC_Os05g02410	Conserved hypothetical protein
	801087	803464	LOC_Os05g02420	expressed protein

	810653	810008	LOC_Os05g02430	retrotransposon protein, putative, Ty3-gypsy subclass
RM10/qGY7.1	22182883	22183862	LOC_Os07g37020	expressed protein
	22184376	22187030	LOC_Os07g37030	cytochrome b6-f complex iron-sulfur subunit, chloroplast precursor
	22189082	22187290	LOC_Os07g37040	PB1 domain containing protein
	22198211	22197477	LOC_Os07g37045	expressed protein
	22200415	22202130	LOC_Os07g37050	retrotransposon protein
	22203598	22202993	LOC_Os07g37060	expressed protein
SNPID457/qGY8.1	19547321	19549026	LOC_Os08g31580	ethylene-responsive transcription factor
	19551008	19551298	LOC_Os08g31590	expressed protein
	19556162	19557020	LOC_Os08g31600	expressed protein
	19559110	19560474	LOC_Os08g31610	hypothetical protein
SNPID202/qGY11.1	24325126	24323882	LOC_Os11g40700	transposon protein, putative, CACTA, En/Spm sub-class
